# The FLOTAC basic technique as a new extraction method for root-knot nematodes (*Meloidogyne* spp.) from soil and roots

**DOI:** 10.3389/fpara.2022.1000673

**Published:** 2022-09-14

**Authors:** Alberto Troccoli, Giada d’Errico, Trifone D’Addabbo, Nicola Sasanelli, Antonio Bosco, Maria P. Maurelli, Laura Rinaldi, Giuseppe Cringoli

**Affiliations:** ^1^ Institute for Sustainable Plant Protection, National Research Council (CNR), Bari, Italy; ^2^ Department of Agricultural Sciences, University of Naples Federico II, Portici, Italy; ^3^ Formerly at Institute of Sustainable Plant Protection, CNR, Bari, Italy; ^4^ Department of Veterinary Medicine and Animal Production, University of Naples Federico II, Naples, Italy; ^5^ Department of Veterinary Medicine and Animal Production, University of Naples, Regional Centre for Monitoring of Parasitosis (CREMOPAR), Eboli, Italy

**Keywords:** phytoparasitic nematodes, extraction methods, centrifugal flotation, FLOTAC, Root maceration

## Abstract

FLOTAC Techniques have been widely acknowledged as an effective method for the extraction of human and animal parasites. The present study is the first application of FLOTAC basic technique (FBT) for the extraction of phytoparasitic nematodes from soil and infested plant roots. Eggs and second stage juveniles (J_2_) of the root-knot nematode *Meloidogyne incognita* were extracted from infested soil and tomato roots either by FBT and conventional nematode extraction methods, such as centrifugal flotation and root maceration techniques, respectively. The number of *M. incognita* J_2_ and eggs extracted from soil by FBT was always significantly higher compared to the extraction with the centrifugal flotation method, averaging 277 *vs* 35 eggs and J_2_ mL^-1^ soil. Conversely, no significant differences were observed between FBT and the root maceration technique in the extraction of eggs and J_2_ from tomato roots. Results demonstrated that FBT can be highly effective also for the extraction of phytoparasitic nematodes. Due to its accuracy and sensitivity, FBT seems particularly suitable for nematode surveys in wide geographical areas, where an accurate and rapid detection of present phytoparasitic nematofauna is required.

## 1 Introduction

Plant parasitic nematodes are recognized as a major threat to world agriculture, as causing heavy crop yield losses, estimated as exceeding 157 billion dollars/year at world level (Nicol et al., 2011). Most of these losses are due to root-knot nematodes of the genus *Meloidogyne* Goeldi, as highly destructive pests of a wide range of herbaceous and woody crops (Jones et al., 2013). These damages are expected to furtherly increase in the future, due to world economic globalization and climate changes, which will favor the introduction of alien species and the intensification of attacks of endemic pests (Gregory et al., 2009). In this scenario, monitoring soil phytoparasitic nematode populations is necessary either to minimize the impact of alien species on agricultural ecosystems, and to prevent a further spread of endemic crop nematode pests (Singh et al., 2013).

A careful assessment of soil phytonematode population densities is needed to predict the potential yield losses and to select appropriate management strategies (Seinhorst, 1986; Sasanelli et al., 2018; Sasanelli et al., 2021). An accurate quanti-qualitative assessment of soil nematophauna is also basic to ecological studies, as soil nematodes are reliable bioindicators of environmental impact of biotic and abiotic stress factors (Bongers and Ferris, 1999; Du Preez et al., 2022). Based on the above considerations, the choice of effective and accurate nematode extraction techniques is a key point for scientific studies as well as for extension services.

Nematode extraction methods vary according to substrates to process (soil or plant tissues), nematode size, feeding habitus (ecto- or endoparasite) and biology (free living, root-knot or cyst-forming species). Extraction techniques include decanting and sieving, incubation and centrifugal flotation procedures (**Table 1**) (Hooper et al., 2005). These methods are often combined to overcome weaknesses of each individual technique. Cobb’s sieving and decanting method (Cobb, 1918) and the Baermann’s funnel incubation (Baermann, 1917) methods have a general application for nematode extraction from soil samples, while Coolen’s centrifugal flotation method (Coolen, 1979) and the Hussey and Barker’s root maceration technique (Hussey and Barker, 1973) are more frequently used for the extraction of different stages (juveniles, adults and eggs) of root knot nematodes (*Meloidogyne* species) from soil and roots, respectively. In addition, the Fenwick’s can method (Fenwick, 1940) is specifically addressed to the extraction of egg-containing cysts of cyst-nematode species (*Heterodera* spp, *Globodera* spp). All these techniques are efficient but are time and labour consuming, as not allowing routine use in laboratories processing large amounts of samples per day or a direct analysis of samples in field.

**Table 1:** Nematodes extraction methods.

Method	Plant material (roots, leaves, wood)	Soil
Decanting and sieving (Cobb, 1918; Hooper et al., 2005)		X
Incubation – Baermann’s funnels (Baermann, 1917; Hooper et al., 2005)	X	X
Centrifugal flotation (Coolen, 1979)		X
Maceration - centrifugation (Hussey and Barker, 1973)	X	
Flotation by Fenwick can (Fenwick, 1940)		X

**Figure 1:**
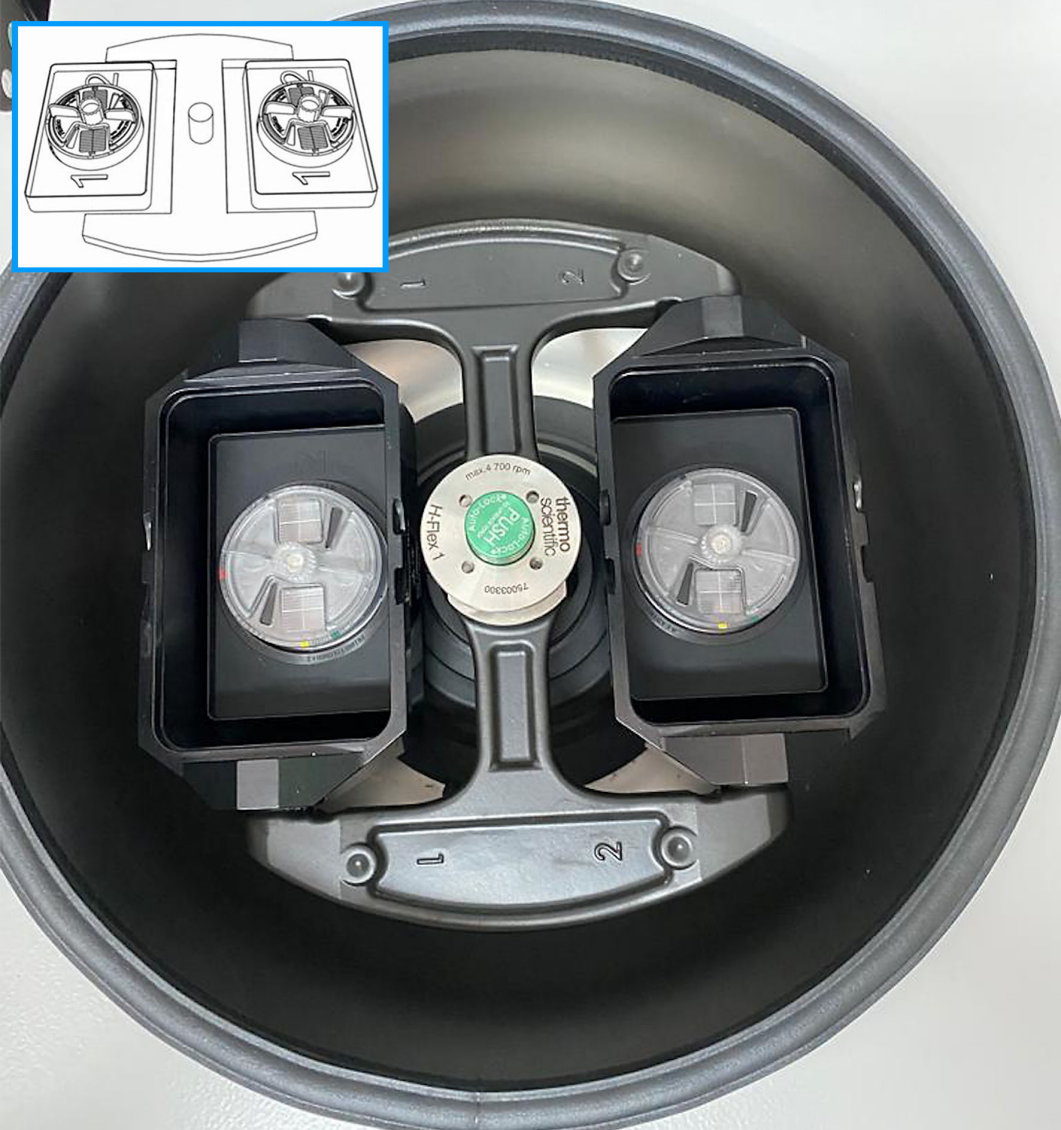
FLOTAC apparatus in a benchtop centrifuge with rotor for microtitre plates. The insert shows a schematic drawing of FLOTAC devise.

In the last two decades, Cringoli et al. (2010) developed and validated a quali-quantitative techniques (FLOTAC basic, dual and double techniques) for a rapid detection and count of parasitic elements (cysts, oocysts, eggs and larvae) in animals and humans. These methods make use of the FLOTAC apparatus, a cylindrical device with two 5-ml flotation chambers (**Figure 1**). The FLOTAC techniques are based on centrifugal flotation of sample followed by removal of floating suspension and have been repeatedly proven as multivalent, sensitive, specific, accurate, precise and highly reproducible, recently demonstrating an optimal efficiency also for investigating the presence of parasitic elements in fresh salad and fruits (Barlaam et al., 2021; Cringoli et al., 2021; Barlaam et al., 2022). Due to its technical characteristics, FLOTAC could be potentially used also for the extraction of eggs and other life stages of phytoparasitic nematodes both from plant roots and soil. The aim of this study was to evaluate the efficiency of FLOTAC basic technique (FBT) for the extraction of root-knot nematodes from soil and plant roots, in comparison with two methods currently used in nematology labs, i.e. the centrifugal flotation and the root maceration technique, respectively.

## 2 Materials and methods

### 2.1 Nematode population

An Italian population of the root-knot nematode *Meloidogyne incognita* (Kofoid and White) Chitw, previously identified by morphometrical parameters, was reared on tomato (*Solanum lycopersicum* L.) cv Regina di Fasano for two months in a glasshouse at 25 ± 2°C. The infested tomato roots were minutely comminuted, thoroughly mixed and then used to artificially infest soil or directly addressed to the comparison of root extraction techniques.

### 2.2 Preparation of soil and root samples

The infested sandy soil (pH 7.2; sand >98%; silt <1%; clay <1%; organic matter = 0.75%) from the glasshouse multiplication of *M. incognita* was thoroughly mixed in a concrete mixer and enriched with the infested root inoculum. The soil mixture was then divided into twenty-four 500 mL samples used for the comparative evaluation of the two extraction methods (Coolen’s method *vs* FLOTAC basic technique).

The infested tomato roots were subdivided into 24 samples of about 5 g each used for eggs and juveniles extraction by the two methods in comparison.

### 2.3 Nematodes extraction from soil

#### 2.3.1 Extraction by the Coolen’s method

**Figure 2:**
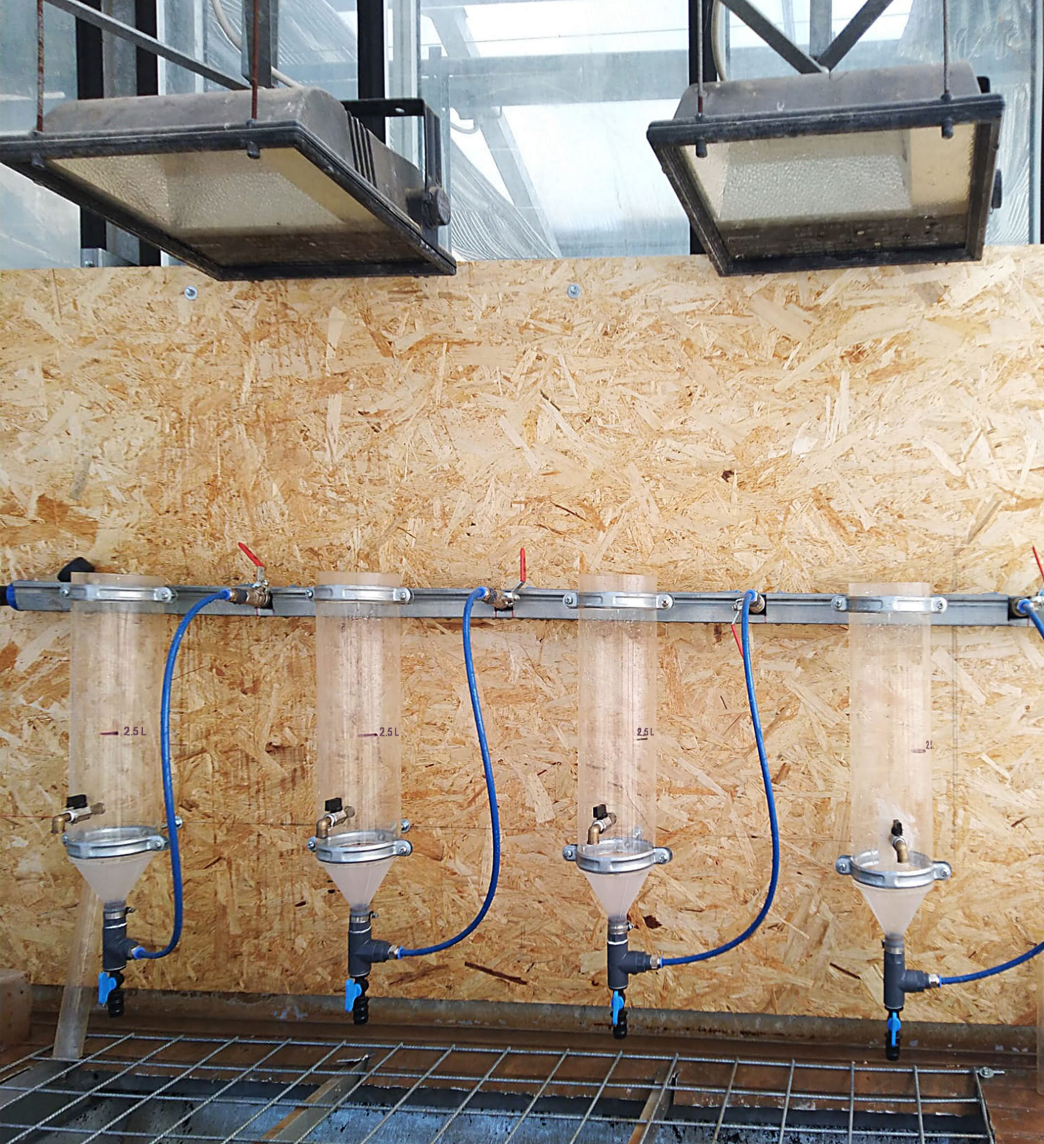
Coolen apparatus.

Three series of four 500 mL soil samples were thoroughly stirred in four buckets with 1 L of water each and filtered through a 2 mm sieve, recovering each soil suspension in a tube of the Coolen’s apparatus (**Figure 2**). In the extraction from soil, infested by the root-knot nematode *M. incognita*, the addition of 100 mL of a 5% sodium hypochlorite water solution and few mL of a wetting agent (silicon antifoam 2%) was also provided, in order to dissolve nematode egg mass gelatinous matrix. Tubes were then filled with water up to a 2.5 L volume and stirred by pressured air for 5 min. A 250 mL volume of the soil suspension was then collected and poured into a metallic centrifuge tube, previously added with 5 mL of kaolin powder, and then filled with water up to a 0.5 L volume and thoroughly stirred. The four metallic tubes were then centrifuged at 2,000 rpm for 5 min, after which the supernatant was discarded. The gelatinous residue retained on the bottom of the tube was re-suspended with 400 mL of a magnesium sulphate solution (1.165 specific gravity), stirred and newly centrifuged at 2,000 rpm for 5 min. The supernatant was then poured into plexiglass 22 μm sieve and eggs and J_2_ were recovered for microscopical count. Nematodes were counted using a stereo microscope at 20x magnification and their total number was recorded.

#### 2.3.2 Extraction by the FLOTAC basic technique - soil

**Figure 3:**
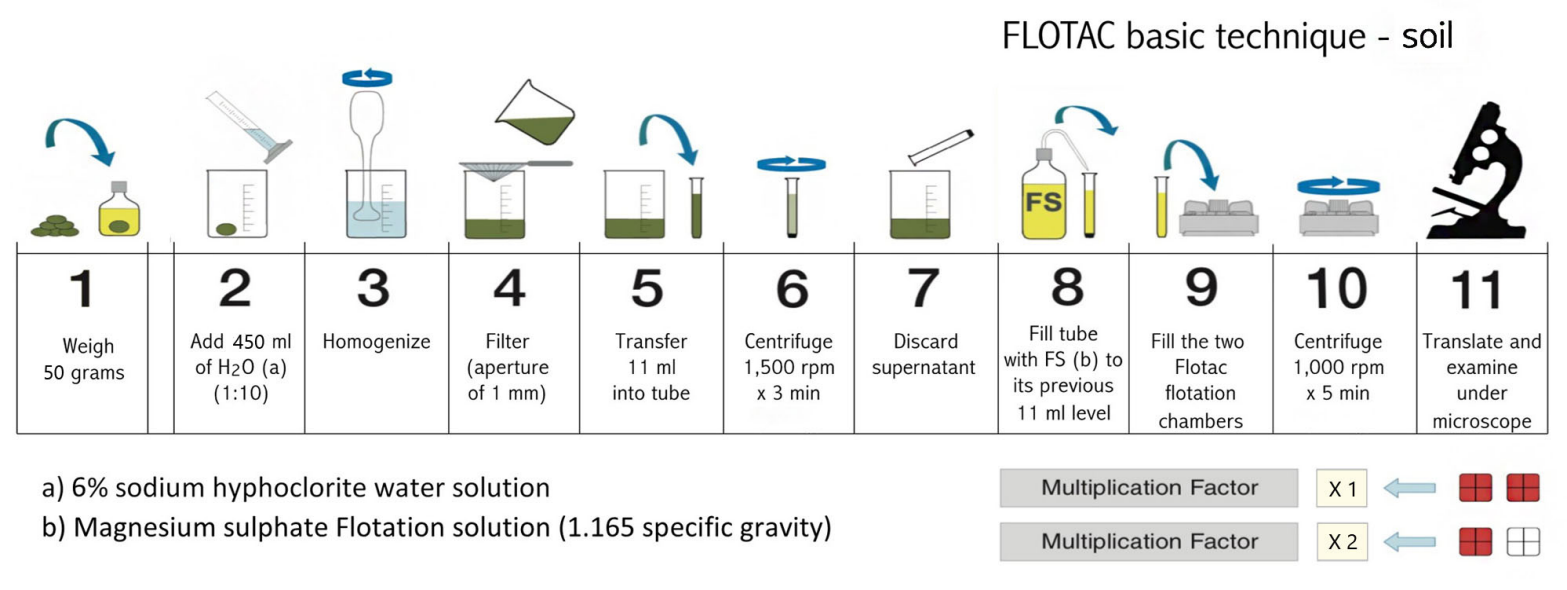
Schematic drawings showing the 11 operating steps of FLOTAC basic technique for the extraction of nematode from soil.

The 11 operating steps of the FLOTAC basic technique are summarized in **Figure 3**. Soil sample was sieved with a wire mesh (aperture of 2 mm), then 50 g were weighed. A 6% sodium hypochlorite water solution (450 mL) was added to the 50 g of soil to reach a volume of 500 mL (dilution ratio 1:10). The suspension was thoroughly homogenized (the use of a hand blender is suggested) and filtered through a wire mesh (aperture of 1mm). One aliquot of 11 mL of the filtered suspension was transferred into a conic tube. The tubes were centrifuged for 3 min at 170 g (1,500 rpm) at room temperature and after centrifugation, the supernatant was discarded, leaving only the sediment (pellets) in the tube. The tubes were filled with a magnesium sulphate solution (1.165 specific gravity) (Cringoli et al., 2010) up to the previous 11 mL level. The suspensions were thoroughly homogenized (before and between the filling) and the two flotation chambers of the FLOTAC apparatus (**Figure 1**) were filled. The FLOTAC apparatus was centrifuged for 5 min at 120 g (1,000 rpm) at room temperature, translated and examined under an optical microscope. The detection limit of this protocol (and multiplication factor) was 1 egg or J_2_ per gram of soil.

### 2.4 Nematode extraction from roots

#### 2.4.1 Extraction by the Hussey and Barker’s method

**Figure 4:**
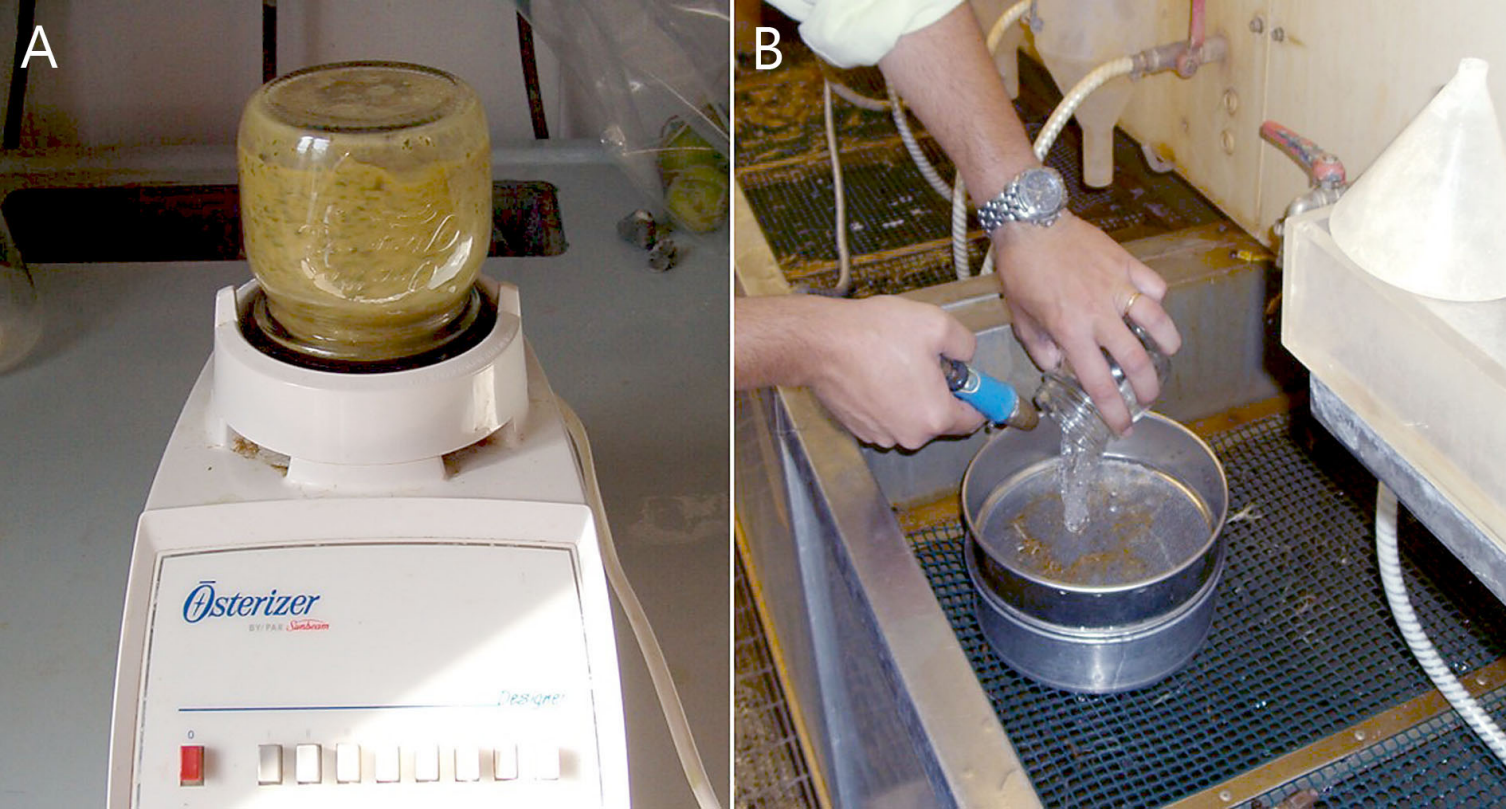
Extraction of eggs and J_2_ from roots by Hussey and Barker’s method. **(A)** Blender containing the root suspension; **(B)** water suspension containing roots poured through the 250 μm (top) and 22 μm (bottom) pore sieves.

Each root sample was grinded for 30 s in a blender containing 150 mL of a 1% aqueous sodium hypochlorite solution (**Figure 4A**). The water suspension was then sieved through a 250 μm and a 22 μm pore sieves (**Figure 4B**). Nematodes and root debris recovered on the 22 μm pore sieve were further centrifuged at 2,000 rpm for 5 min in 400 mL of a magnesium sulphate solution (1.165 specific gravity). The supernatant suspension was then poured onto a 22 μm pore sieve and eggs and J_2_ were recovered for microscopical count.

#### 2.4.2 Extraction by the FLOTAC basic technique

**Figure 5:**
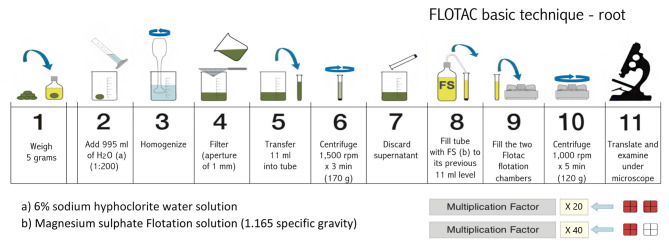
Schematic drawings showing the 11 operating steps of Flotac basic technique for the extraction of nematode from roots.

The 11 operating steps of the FLOTAC technique are summarized in **Figure 5**. Five grams of root sample were placed in the becker of a blender and a 6% sodium hypochlorite water solution was added up to the volume of 200 ml (a drop of a wetting agent was added) and grinded thoroughly. The suspension was filtered through a wire mesh (aperture of 1 mm) and another part of 6% sodium hypochlorite water solution (800 mL) was added, rinsing the blender and wire mesh, in order to obtain a final dilution ratio of 1:200. One aliquot of 11 mL of the filtered suspension was transferred into a conic tube and the same steps of the protocol previously described for soil samples were applied. Using this protocol, the detection limit (multiplication factor) was 20 eggs or J_2_ per gram of root.

### 2.5 Statistical analysis

All the 12-sample soil and root extraction series were repeated twice and data from the two experimental runs were pooled in the absence of a significant experiment per treatment interactions (Finney, 1978). Data from the two extraction methods were statistically analyzed by analysis of variance (ANOVA) and means compared by the Student’s *t* test (P = 0.01). Statistical analyses were performed using the software Plot IT Vers. 3.2 (Scientific Programming Enterprises, Haslett, Mi, USA).

## 3 Results

The numbers of *M. incognita* J_2_ and eggs extracted from soil by FLOTAC basic technique (FBT) were always significantly higher compared to the extraction with the centrifugal flotation method (**Table 2**). In particular, FBT extracted a mean of 277 eggs and J_2_ mL^-1^ soil versus 35 eggs and J_2_ mL^-1^ soil recovered by centrifugal flotation, i.e. an 8-fold higher number. By contrast, no statistical differences occurred between the two methods in the extraction from tomato roots, averaging 20,361 and 20,474 eggs and j_2_ g^-1^ root for FBT and maceration-centrifugation, respectively, despite the presence of an aberrant replication in FBT data (9,423 eggs and J_2_ g^-1^ root) (**Table 2**). Values of Standard Deviation (SD) and Coefficient of Variation (CV) indicated a low variability of data among the replications from both methods.

**Table 2:** Statistical comparison of number of *M. incognita* eggs and J_2_ recovered from 12 soil and 12 root samples extracted by the considered methodologies (Coolen’s method for soil; Hussey and Barker for roots and FLOTAC basic technique for soil and roots).

Sample(replications)	Soil(N° eggs and J_2_ mL^-1^ soil)		Root(N° eggs and J_2_ g^-1^ root)	
	Centrifugal flotation	FLOTAC basic technique	Maceration-centrifugation	FLOTAC basic technique
1	51.8	324	21,548	18,917
2	49.3	322	21,262	9,423
3	33.3	267	21,357	21,050
4	25.3	260	20,671	26,610
5	40.7	258	24,540	24,220
6	30.0	311	19,700	23,340
7	32.0	233	11,310	19,678
8	37.3	271	17,942	19,944
9	34.7	267	28,261	20,207
10	36.0	222	18,809	20,159
11	24.0	319	27,614	21,042
12	28.7	277	12,673	19,740
Mean	35.3	277.6^**^	20,474	20,361^ns^
SD	8.6	34.4	5,093	4,118
CV	24.4	12.4	24.9	20.2

**Statistically different from centrifugal flotation according to Student's *t * test (P = 0.01);

ns, no statistically different from Maceration/centrifugation; SD, standard deviation;

CV, coefficient of variation.

## 4 Discussion

The efficiency of any technique for nematode extraction from soil and plant materials results from a sum of parameters, such as sensitivity, accuracy and reproducibility of results (McSorley and Walter, 1991). High sensitivity and accuracy of the extraction method is essential to a prompt diagnosis of nematode infestation also in the presence of low population levels. Sensitive and accurate extraction tools are also crucial in ecological studies aimed to detect shift in composition of soil nematophauna related to biotic or abiotic disturbance factors. Reproducibility of extraction yield can ensure the comparability of nematode population data either throughout the time and among different treatments in comparison.

FLOTAC basic technique has been previously acknowledged as an effective method for a rapid and reliable diagnosis of human and veterinary infectious and parasitic diseases, due to its high sensitivity, precision and accuracy (Knopp et al., 2011; Levecke et al., 2012; Steinmann et al., 2012; Ramos et al., 2016).

In our experiments, FBT was demonstrated to be more effective than the centrifugal flotation method for the extraction of *M. incognita* J_2_ and eggs from soil, whereas its efficiency was not consistently higher in nematode extraction from roots. Furthermore, nematode suspensions resulting from both soil and root extraction by FBT were clearer and free of debris compared to those obtained with conventional methods, as allowing an easier microscopical observation.

Another plus point of FBT is the need of few laboratory infrastructures, as limited to a benchtop centrifuge and few consumables. Previous parasitological studies reported a rapid and reliable analysis of large numbers of animal samples even in the absence of centrifuges and other basic equipment by an evolution of FLOTAC, namely the mini-FLOTAC (Cringoli et al., 2017; Lozano et al., 2021).

However, extraction efficacy ofFBT is strongly affected by type and density of flotation solution, as largely varying among sucrose, magnesium sulphate and zinc sulphate, i.e. substances most commonly used (Coolen and D’Herde, 1977; Cringoli et al., 2010). In our pivotal experiments (data not showed), zinc sulphate provided a similar extraction efficiency compared to magnesium sulphate, but severely affected J_2_s morphology and, consequently, their microscopical identification in the presence of saprophytic nematode specimens.

Another factor to be considered is the soil composition. In our study, experiments were performed in sandy soil. However, as recovery of nematodes might show different results in detection based on the type of soil (*i.e.* clay, silty, silty-loamy, etc.), evaluation of Flotac extraction performance also in soil with different grain size should be assessed.

Based on the above considerations, FBT is strongly suggested for processing large number of soil samples from nematode surveys in wide geographical areas, where all the occurring phytoparasitic nematodes should be accurately detected. Other fields of application of FLOTAC could be represented by ecological studies, as requiring detection of the whole soil nematophauna, as well as by the extraction of entomopathogenic nematodes from insects for their application as biocontrol agents. Conversely, the huge amounts of root and soil samples usually coming from nematode control experiments can be more easily managed with the conventional extraction techniques, which allow to process more samples at the same time.

## Data availability statement

The original contributions presented in the study are included in the article/supplementary material. Further inquiries can be directed to the corresponding author.

## Author contributions

AT, TD, NS and GC designed the experiments. TD, NS, AB and GC analyzed the data. AT, TD, NS and GC drafted the manuscript with input from all the co-authors. AT, NS, Gd'E, AB and GC performed extraction experiments and microscopy analysis. GC supervised the project and arranged the funding. All authors contributed to the article and approved the submitted version.

## Funding

The work was financially supported by the CREMOPAR.

## Acknowledgment

Authors thanks Mr. Salatino N. and Mr. Catalano F. of IPSP-CNR for their technical assistance.

## Conflict of interest

The authors declare that the research was conducted in the absence of any commercial or financial relationships that could be construed as a potential conflict of interest.

## Publisher’s note

All claims expressed in this article are solely those of the authors and do not necessarily represent those of their affiliated organizations, or those of the publisher, the editors and the reviewers. Any product that may be evaluated in this article, or claim that may be made by its manufacturer, is not guaranteed or endorsed by the publisher.
